# Comprehensive toxicological, metabolomic, and transcriptomic analysis of the biodegradation and adaptation mechanism by *Achromobacter xylosoxidans* SL-6 to diuron

**DOI:** 10.3389/fmicb.2024.1403279

**Published:** 2024-06-07

**Authors:** Zhixu Hu, Cancan Qian, Haodong Wang, Lanlan Sun, Cailan Wu, Guoqiang Zhang, Xiaoqiang Han, Chunjuan Wang, Ting Ma, Desong Yang

**Affiliations:** College of Agriculture/Key Laboratory of Oasis Agricultural Pest Management and Plant Protection Resources Utilization, Shihezi University, Shihezi, China

**Keywords:** *Achromobacter xylosoxidans*, degradation, bioremediation, diuron, multiomics

## Abstract

Biodegradation was considered a promising and environmentally friendly method for treating environmental pollution caused by diuron. However, the mechanisms of biodegradation of diuron required further research. In this study, the degradation process of diuron by *Achromobacter xylosoxidans* SL-6 was systematically investigated. The results suggested that the antioxidant system of strain SL-6 was activated by adding diuron, thereby alleviating their oxidative stress response. In addition, degradation product analysis showed that diuron in strain SL-6 was mainly degraded by urea bridge cleavage, dehalogenation, deamination, and ring opening, and finally *cis, cis*-muconic acid was generated. The combined analysis of metabolomics and transcriptomics revealed the biodegradation and adaptation mechanism of strain SL-6 to diuron. Metabolomics analysis showed that after the strain SL-6 was exposed to diuron, metabolic pathways such as tricarboxylic acid cycle (*cis, cis*-muconic acid), glutathione metabolism (oxidized glutathione), and urea cycle (arginine) were reprogrammed in the cells. Furthermore, diuron could induce the production of membrane transport proteins in strain SL-6 cells and overexpress antioxidant enzyme genes, finally ultimately promoting the up-regulation of genes encoding amide hydrolases and dioxygenases, which was revealed by transcriptomics studies. This work enriched the biodegradation mechanism of phenylurea herbicides and provided guidance for the removal of diuron residues in the environment and promoting agriculture sustainable development.

## Graphical Abstract

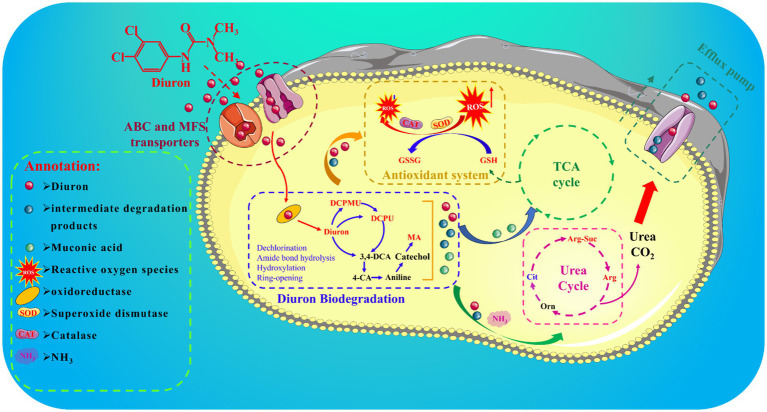

## 1 Introduction

Diuron (3-(3,4 dichlorophenyl)-1,1 dimethylurea) was a phenylurea herbicide that was extensively acted to Broadleaf weeds and grassy weeds in diverse crops fields such as cotton, fruit, and cereals (Li et al., 2021; Wang et al., 2022). Its function was to block the binding sites of plastid quinones in photosynthetic system II, and then destroy electron transport, thereby leading to weed death. However, most studies indicated that diuron was not easily volatile and photolytic in the soil, which could cause phytotoxicity and damage to subsequent sensitive crops, and induce environmental contamination of groundwater (Tandon and Pant, 2019). In addition, the ecological toxicity of diuron on non-target organisms was also reported, thereby raising community worry about its adverse impact on human health (Kao et al., 2019). Previous studies indicated that the diuron could cause endocrine and respiratory system disorders through its carcinogenic, mutagenic, and neurotoxic properties (Rocha et al., 2013; Huovinen et al., 2015; Behrens et al., 2016). Therefore, it is an urgent development for researchers to find a safe and effective method to manage the environmental pollution problem of diuron.

Currently, diuron could be degraded by means of physical adsorption, photocatalysis, chemistry, and microorganisms in the environment (Park and Jhung, 2020). Among them, microbial degradation was a safe and effective method to control organic contaminants from the natural environment (Bhatt et al., 2021). At present, only a few bacterial strains have been reported to degrade diuron, such as *Neurospora intermedia* (Wang et al., 2017), and *Bacillus licheniformis* (Singh and Singla, 2019). However, most of these bacterial isolates were unable to achieve complete mineralization of diuron and ended up producing only 1-(3,4-dichlorophenyl)-3-methyl urea (DCPMU), 1-(3,4-dichlorophenyl) urea (DCPU), or 3,4-dichloroaniline (3,4-DCA). In addition, previous research mainly focused on improving biodegradation efficiency, biodegradation characteristics, and identification of biodegradation products. There were relatively few reports on the resistance and transformation mechanism of microorganisms to diuron (Bhatt et al., 2021). Importantly, multi-omics technology provides new research ideas for elucidating the degradation mechanism of organic contaminants (Manzoni et al., 2018; Chunyan et al., 2023). Transcriptomics was used to explore key genes and degradation principles of microorganisms degrading pollutants (Filiatrault, 2011). Furthermore, metabolomics provided key information for us to further understand related metabolic pathways and their changes in the biodegradation process of aromatic compounds (Gao et al., 2021). Therefore, analyzing transcriptome and metabolome data could help us systematically understand the adaptation and biodegradation mechanisms of bacteria to diuron.

In this study, *Achromobacter xylosoxidans* SL-6 obtained in the early stage was used as the experimental object to investigate the biodegradation mechanism of diuron. Therefore, we combined toxicological evaluation, biodegradation product identification, metabolome analysis, and transcriptome analysis to reveal: (1) the adaptation mechanism of strain SL-6 to the oxidative stress caused by diuron; (2) the transport mechanism of strain SL-6 to diuron in cells; (3) the degradation mechanism of strain SL-6 to diuron in cells.

## 2 Materials and methods

### 2.1 Chemicals and medium

Diuron (analytically pure ≥97.0%) was provided by Shanghai McLean Biochemical Technology Co., Ltd. HPLC grade *n*-hexane and methanol were obtained from Thermo Fisher Scientific, United States. Mineral salt medium (0.2 g MgSO_4_·7H_2_O, 1 g (NH4)_2_ SO_4_, 1.5 g K_2_HPO_4_, 0.5 g NaCl, and 0.5 g KH_2_PO_4_ per litre of water; Sigma-Aldrich; purity ≥99%). Luria-Bertani (LB) medium (10.0 g tryptone, 5.0 g yeast extract, and 10.0 g NaCl per litre of water; Sigma-Aldrich; purity ≥99%). All chemicals were analytical reagent grade.

### 2.2 Biodegradation characteristics of strain SL-6 to diuron

Previously, our research team has successfully discovered a strain that efficiently degrades diuron. The strain was isolated from the agricultural test site of Shihezi University, Shihezi City, Xinjiang Uygur Autonomous Region, China. The strain was identified and named *Achromobacter xylosoxidans* SL-6. Supplementary Figure S1 displays the colony morphology and evolutionary tree of the SL-6 strain. The results of the biological characteristics study of strain SL-6 are shown in Supplementary Figure S2. It was found that when the diuron dose reached 200 mg/L, the biodegradation efficiency of strain SL-6 was 93.1% within 5 days.

### 2.3 Determination of the cytotoxicity of diuron to the strain SL-6

Strain SL-6 was inoculated into MSM medium containing several doses of diuron (0, 50, 100, 200, 400, 600 mg/L) and cultured for 24 h and 48 h, respectively. After washing three times with PBS, the cells were performed at 4°C and 8,000 rpm to obtain bacterial cells. The reactive oxygen species (ROS) level, malondialdehyde (MDA) content, and lactate dehydrogenase (LDH) activity in strain SL-6 were measured, which was described in Supplementary Section SI-1.

### 2.4 Strain SL-6 antioxidant enzyme activity assay

Superoxide dismutase (SOD), catalase (CAT), and glutathione (GSH) were essential antioxidants for maintaining redox homeostasis in organisms (Staerck et al., 2017). The strain SL-6 was inoculated in several doses of diuron (0, 50, 100, 200, 400, 600 mg/L) and cultured for 24 h and 48 h, respectively. The cells were ultrasonically fragmented at 0°C for 15 min and centrifuged at 8,000 rpm to collect the supernatant. The activity of SOD, CAT, and the content of GSH in strain SL-6 cells was detected by the reagent kit (Jiancheng Bioengineering Institute, Nanjing, China).

### 2.5 Degradation of diuron and identification of metabolites

The sample was extracted twice with *n*-hexane and the organic phase was combined. The organic solution was removed by the rotary evaporator and then redissolved with methanol. The degradation products of diuron were detected by GC-MS (Agilent 8,890-5977B, Agilent Technologies, CA, United States). The column temperature program, scan range, and the parameters of the mobile phase are shown in Supplementary Section SI-2.

### 2.6 Metabolomic analysis

To detect alterations in intracellular metabolites, we performed a nontargeted metabolomic analysis (Gika et al., 2019). Strain SL-6 cells grown in LB medium supplemented with 200 mg/L diuron were harvested. The appropriate amount of sample was added to 1,000 μL pre-cooled methanol/acetonitrile/water solution (2:2:1, v/v/v) and treated with a vortex oscillator for 30 s. After ultrasonication at 4°C for 25 min, it was placed at −20°C for 15 min. The samples were placed in a centrifuge at 4°C, 14,000 rpm for 15 min. The supernatant was dried in a vacuum concentrator. Then it was redissolved with 100 μL acetonitrile/water (1:1, v/v) solvent and vortexed for 30 s, and then placed in a 4°C, 14000 rpm centrifuge for 15 min. The extracted samples were detected by UPLC-Q-TOF/MS. The specific parameters of LC-MS/MS analysis were described in Supplementary Section SI-3.

### 2.7 Transcriptome analysis

The strain SL-6 was inoculated into MSM liquid medium containing diuron (200 mg/L) and without diuron by 10% inoculation volume, and three replicates were set up, respectively, (An et al., 2020). The cells were cultured in a shaker at 30°C and 180 rpm for 72 h. Centrifugation was performed at 4°C and 10,000 rpm for 10 min to collect bacterial sediment. Washed twice with PBS, frozen in liquid nitrogen for 5 min, and stored at −80°C. The transcriptome sequencing service of the test samples was carried out by Wuhan Kangce Technology Co., Ltd. (Wuhan, China). The specific process of transcriptome sequencing was described in Supplementary Section SI-4.

### 2.8 Statistical analysis

Statistical analysis was performed using IBM SPSS statistics software (version 26.0, IBM Corporation, New York, United States). The significance of the difference between the treatment group and the control group was compared by single factor analysis of variance and Duncan test. All data were expressed as the means ± standard deviation (SD) at the *p* < 0.05 level. Data visualization was performed using Origin 2023 software (OriginLab, Massachussets, United States).

## 3 Results

### 3.1 Effect of diuron on the antioxidant system of strain SL-6

**Figure 1:**
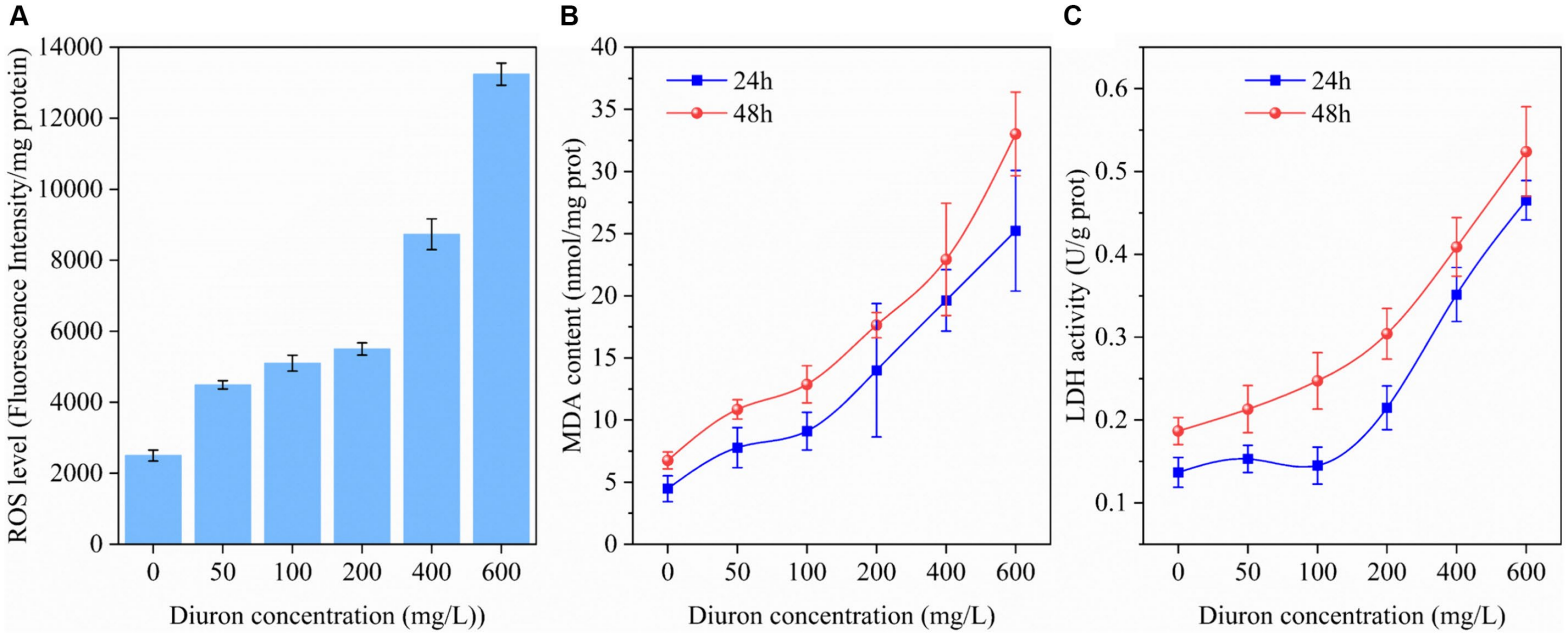
Effects of different concentrations of diuron on **(A)** ROS level (48 h), **(B)** MDA content, and **(C)** LDH activity of *Achromobacter xylosoxidans* SL-6 after treatment for 24 h and 48 h, respectively. Error bars represent standard deviation (*p* < 0.05).

It was reported diuron could induce lipid peroxidation of the cell membrane by generating ROS, thereby leading to oxidative stress injury (Wang et al., 2022). ROS, MDA, and LDH were common indicators of cellular oxidative damage (Wakeel et al., 2020). Therefore, the intracellular ROS, MDA content, and LDH activity of strain SL-6 during treatment with diuron at different initial concentrations were determined. The results indicated that the ROS level, MDA content, and LDH activity of strain SL-6 cells treated with diuron was enhanced in a concentration-dependent manner (Figure 1). In addition, after 600 mg/L diuron treatment for 48 h, the ROS level, MDA content, and LDH activity of cells were increased by 4.29, 3.89, and 1.80 times than that of the control group, respectively. This indicated that diuron could induce the production of ROS in strain SL-6 cells, leading to enhanced lipid peroxidation and ultimately causing cell damage of strain SL-6. Previous studies indicated that SOD, CAT, and GSH were the primary antioxidant active substances in cells and played an important role in scavenging excessive ROS to maintain cellular redox balance (Hernández et al., 2015). Therefore, the SOD, CAT activity, and GSH content in the cells exposed to diuron were measured (Supplementary Figure S3). The results showed that after treatment with 200 mg/L diuron, the SOD, CAT activity and GSH content of strain SL-6 cells increased by 80, 159, and 81%, respectively, compared with the control group. This indicated that the addition of low concentration of diuron (≤200 mg/L) could stimulate the increase of SOD, POD activity, and GSH content in strain SL-6 cells, and reduce the oxidative damage of cells by removing excessive ROS in strain SL-6 cells.

### 3.2 Identification of intermediate degradation products of diuron

**Figure 2:**
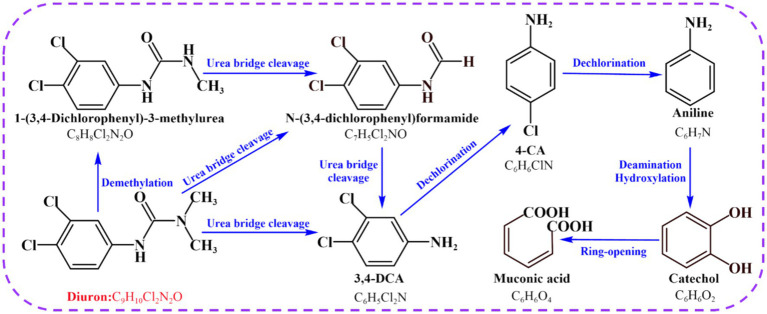
Proposed transformation pathway of diuron by *Achromobacter xylosoxidans* SL-6.

Combined with previous research results, we found that strain SL-6 had the highest biodegradation efficiency to 200 mg/L diuron (Supplementary Figure S2D), and the addition of 200 mg/L diuron had a relatively small effect on the oxidative damage of strain SL-6 cells. Therefore, 200 mg/L was selected as the optimal application dose of diuron in subsequent experiments. At present, the study found that the use of reference mass spectral database is still one of the best approaches to annotate the structure of known metabolites (Vinaixa et al., 2016). A total of seven metabolites were identified according to the mass spectra information by GC/MS detection (Supplementary Figure S4), including demethylation products: DCPMU and DCPU (C_8_H_8_Cl_2_N_2_O and C_7_H_5_Cl_2_NO), amide bond hydrolysis product: 3,4-DCA (C_6_H_5_Cl_2_N), dechlorination products: 4-CA and aniline (C_6_H_6_ClN and C_6_H_7_N), and hydroxylation products: catechol and *cis, cis*-muconic acid (C_6_H_6_O_2_ and C_6_H_6_O_4_). The results confirmed that a series of continuous transformations of diuron occurred in the cells of strain SL-6, including demethylation, urea bridge cleavage, dehalogenation, deamination, hydroxylation, and ring-opening (Figure 2).

### 3.3 Metabolomic analysis of intracellular metabolites of strain SL-6

**Figure 3:**
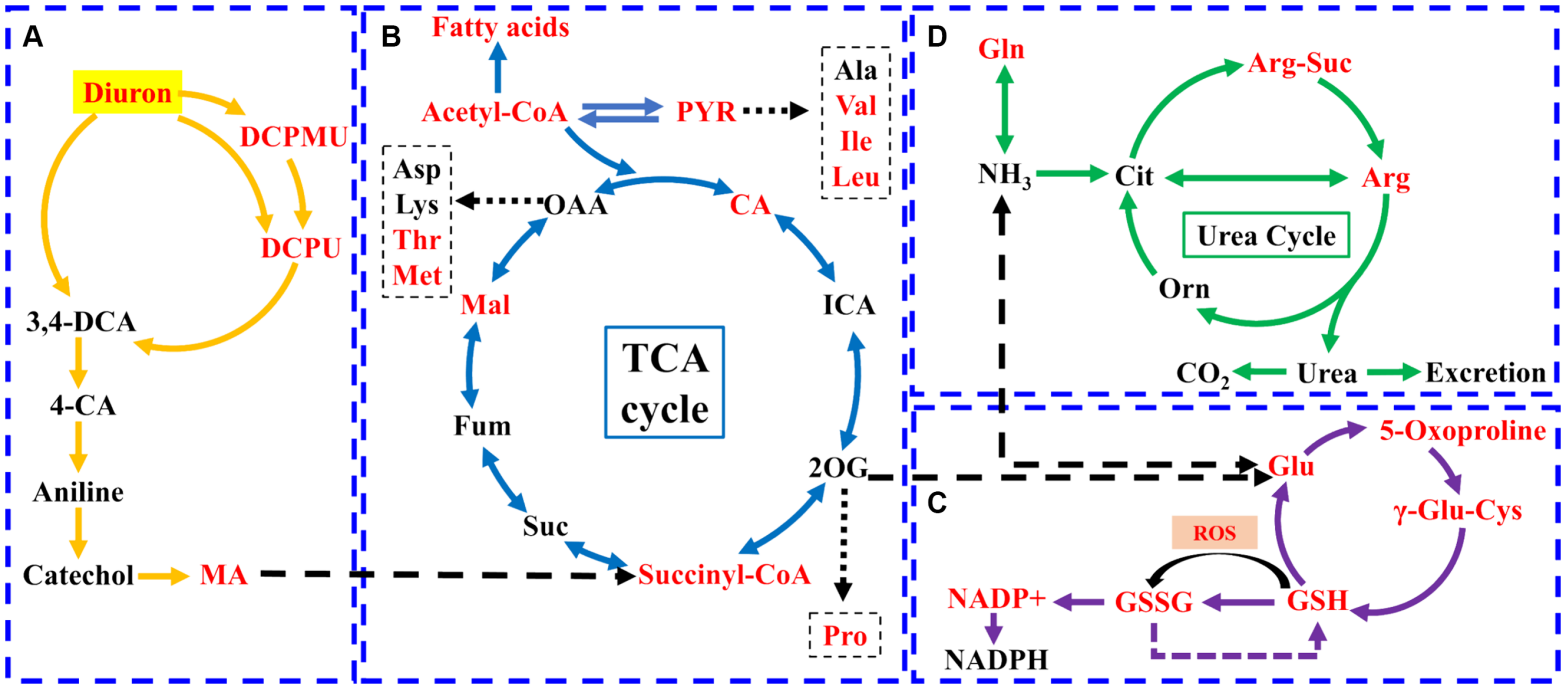
Schematic diagram of the metabolic pathways of strain SL-6 cells exposed to 200 mg/L diuron. **(A)** Diuron degradation pathway. **(B)** TCA cycle. **(C)** Glutathione metabolism. **(D)** Urea cycle. Red represents upregulation. Gln, glutamine; Glu, glutamic acid; Orn, ornithine; Cit, citrulline; Arg-Suc, L-argininosuccinic acid; γ-Glu-Cys, γ-L-glutamyl-L-cysteine; DCPMU, 1-(3,4-dichlorophenyl)-3-methyl urea; DCPU, 1-(3,4-dichlorophenyl) urea; MA, cis, cis-muconic acid; CA, citric acid; ICA, isocitrate; PYR, pyruvate; 2-OG, 2-Oxopentanoic acid; Suc, succinic acid; Fum, fumarate; Mal, malic acid.

Metabolomics method was used to analyze the changes of intracellular metabolites of the strain SL-6 after diuron treatment. The results showed that 1,338 metabolites were detected and analyzed. Principal component analysis (PCA) revealed a clear distinction between the diuron treatment group and the control group on PC1, which represented 79.3% of the total variance (Supplementary Figure S5A). This indicated that the changes in endogenous metabolites of the degrading bacteria SL-6 cells were caused by diuron. Further analysis of metabolites was performed to clarify the differences in metabolites among the two groups. Among them, 388 were significantly up-regulated and 172 were significantly down-regulated (Supplementary Figure S5B). In addition, VIP score plots of relevant metabolites were obtained by PLS-DA (Supplementary Figure S5C). It is worth noting that the addition of 200 mg/L diuron had effects on a variety of metabolites related to diuron degradation or antioxidants by strain SL-6, such as DCPMU, DCPU, malic acid, arginine, eta-carotene, phosphatidylethanolamine, citric acid, and glutathione. Furthermore, KEGG pathways indicated that the metabolic pathways mainly affected by diuron treatment were the biosynthesis of amino acids, 2-oxocarboxylic acid metabolism, alanine, aspartate and glutamate metabolism, and arginine biosynthesis (Supplementary Figure S5D). The above results showed that strain SL-6 could efficiently degrade diuron by the diuron biodegradation pathway, the TCA cycle, glutathione metabolism, and the urea cycle (Figure 3).

There were three degradation products involved in the biodegradation pathway of diuron, namely DCPMU, DCPU, and *cis, cis*-muconic acid (MA) (Figure 3A). The metabolites involved in the TCA cycle were divided into two categories: organic acids and amino acids (Figure 3B). Among them, the contents of succinyl coenzyme A, malic acid, and citric acid increased significantly, and their relative abundance increased by 32.15, 3.60, and 17.78%, respectively, compared with the control group (Supplementary Figure S6). The contents of arginine, glutamic acid, methionine, isoleucine, valine, proline, and threonine were up-regulated (Supplementary Figure S6). This suggested that the TCA cycle could provide energy and various nutrients required for the strain SL-6 to adapt and degrade diuron, and was the center of the degradation metabolic reaction. In addition, the levels of glutamic acid, oxoproline, glutathione, and oxidized glutathione (GSSH) involved in glutathione metabolism were increased, and their relative abundances increased by 12.45, 13.39, 20.43, and 29.72%, respectively, compared with the control group (Figure 3C; Supplementary Figure S6). At the same time, the content of arginine and arginine succinic acid involved in the urea cycle were significantly up-regulated, and their relative abundances increased by 24.59 and 36.53%, respectively, compared with the control group (Figure 3D; Supplementary Figure S6). This indicated that strain SL-6 could reduce the oxidative stress induced by diuron through glutathione metabolism and urea cycle pathway, enhance the detoxification ability of diuron cytotoxicity, and better serve the degradation of diuron.

### 3.4 Transcriptome enrichment analysis of DEGs

**Figure 4:**
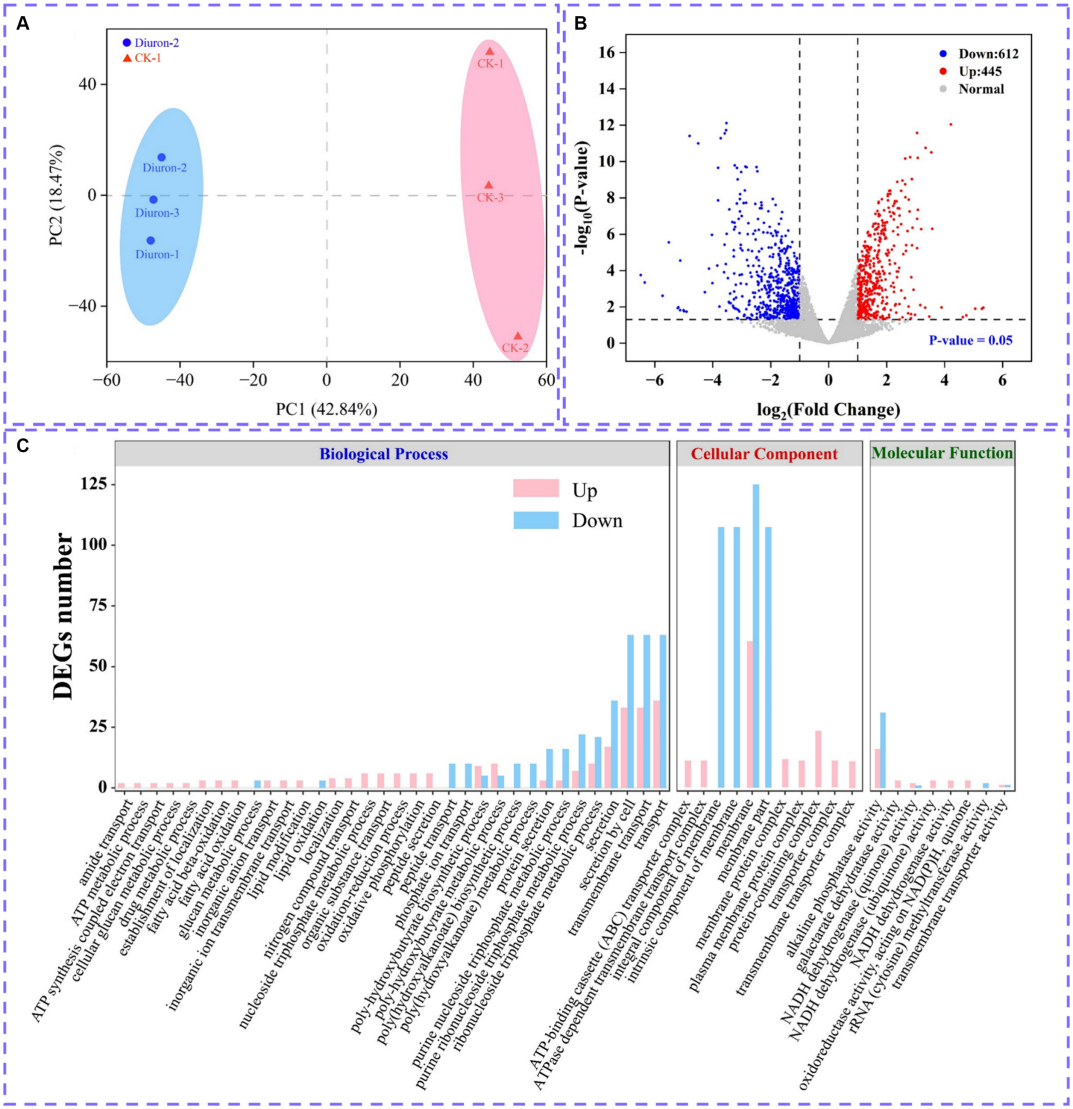
**(A)** PCA analysis plot. **(B)** Volcano plot of all differentially expressed genes. **(C)** Significantly enriched GO terms in differentially expressed genes.

**Figure 5:**
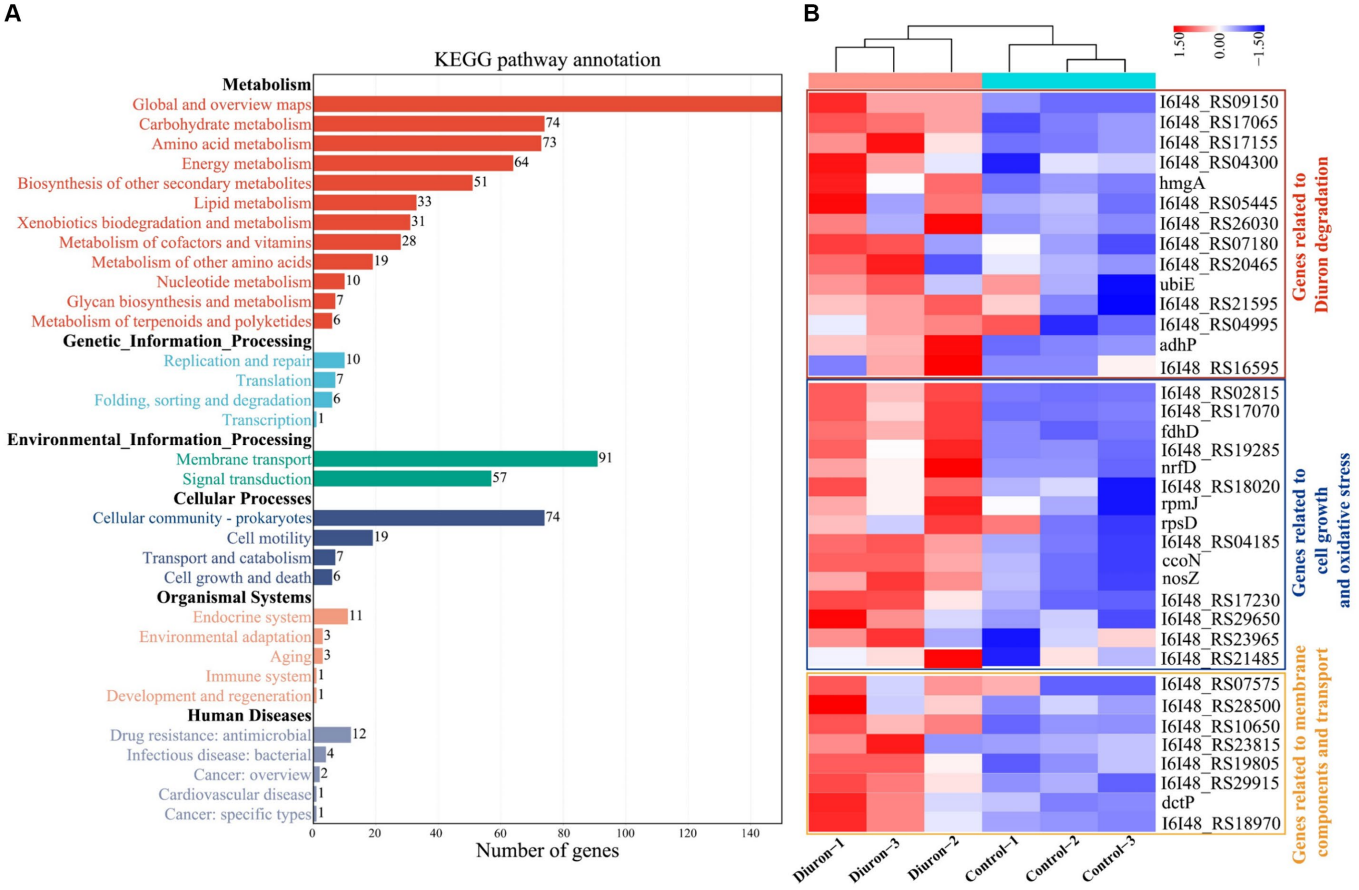
**(A)** KEGG secondary classification of differentially expressed genes. **(B)** Cluster heat map of differentially expressed genes related to transmembrane transport of substances, antioxidant reactions, and the action of diuron-degrading enzymes.

Illumina MiSeq sequencing technology was used to research the differentially expressed genes during the degradation of diuron by strain SL-6, and the biodegradation mechanism of diuron was discussed. The PCA analysis showed that the diuron treatment group (200 mg/L) was significantly separated from the control group along PC1, which explained 42.84% of the total variance (Figure 4A). The results of the volcano plot showed that compared with the control group, the total number of differentially expressed genes (DEGs) after diuron induction was 1,057, of which 445 were up-regulated and 612 were down-regulated (Figure 4B). The 1,057 DEGs were annotated by the GO database and divided into three categories: molecular function, cellular component, and biological process (Figure 4C). In the molecular function classification, some up-regulated genes were annotated as oxidoreductase, dehydrogenase, cytochrome P450 and hydrolase activities (Ma et al., 2023). These DEGs involving the above GO entries might participate in the degradation of diuron by strain SL-6. The most important GO term in the classification of cellular components was ATPase-dependent transmembrane transport complex, followed by transmembrane transport protein complex, plasma membrane protein complex, and transport complex. These DEGs involved in the transmembrane transport of substances might help strain SL-6 transport diuron into cells. In the classification of biological processes, the up-regulated genes were mainly concentrated in carbon metabolism, nitrogen metabolism, fatty acid metabolism, and TCA cycle, while the down-regulated genes focused on cellular carbohydrate metabolism, glucan metabolism, polysaccharide metabolism, and glycogen metabolism (Qi et al., 2023). The above genes might participate in the energy metabolism process in strain SL-6 cells, providing energy to degrade and adapt to diuron. Similar results were observed for KEGG annotated pathway distribution. In KEGG pathway analysis, DEGs were mainly concentrated in multiple metabolic items, such as oxidative phosphorylation, glyoxylic acid and dicarboxylic acid metabolism, nitrogen metabolism, transmembrane transport, the TCA cycle, and glycolysis (Figure 5A).

### 3.5 DEGs related to strain SL-6 adaptation and degradation of diuron

The degradation of diuron in strain SL-6 cells involved a variety of biological processes, including material transmembrane transport, antioxidant response, and the role of diuron degradation enzymes. Hierarchical clustering analysis was performed on DEGs related to the above biological processes (Figure 5B).

Cell transmembrane transport system: genes encoding transmembrane proteins, ATP-binding cassette (ABC), and major facilitator superfamily (MFS) transporters. According to the transcriptome results, we found that the expression of the genes (I6I48_RS07135 and *livG*) encoding Lipopolysaccharide export system ATP-binding protein LptB synthesis were significantly increased by 3.80-fold and 3.34-fold than that of the control group, respectively (Supplementary Table S1). The genes encoding outer membrane protein (I6I48_RS11310, *adeC*, and I6I48_RS00580) were up-regulated by 3.01-fold, 2.99-fold, and 3.19-fold than that of the control group, respectively (Supplementary Table S1). The expressions of nine ABC transporters (I6I48_RS03180, I6I48_RS10650, I6I48_RS28500, I6I48_RS29915, I6I48_RS19805, I6I48_RS18970, I6I48_RS23815 *dctP*, and *pstS*) and one MFS transporters (I6I48_RS22785) were significantly up-regulated (Supplementary Table S1). In summary, we speculate that the strain SL-6 cells had a transmembrane transport system capable of transporting exogenous organic substances, which could transport diuron into the cells to complete the next step of degradation.

Antioxidant system: genes encoding catalase, peroxidase, glutathione metabolism, and redox homeostasis. According to the transcriptome of diuron-treated cells, the genes encoding peroxidase (I6I48_RS30485, I6I48_RS29650, and I6I48_RS11345) and catalase (I6I48_RS02815) were significantly up-regulated (Supplementary Table S2). The genes encoding glutathione S-transferase (I6I48_RS26100) and glutaredoxin (I6I48_RS17070) were up-regulated by 2.34-fold and 2.47-fold than that of the control group, respectively (Supplementary Table S2). Three cellular redox homeostasis-related genes (I6I48_RS18035, I6I48_RS30485, and I6I48_RS05390) were up-regulated by 2.05-fold, 2.48-fold, and 2.57-fold than that of the control group, respectively (Supplementary Table S2). Taken together, we speculate that there was an antioxidant system in the cells of strain SL-6 that could resist exogenous toxic substances and help the strain cells maintain normal survival, so as to ensure the degradation of diuron.

Diuron degradation system: genes encoding demethylase, amidase, amidohydrolase, dehalogenase, monooxygenase and dioxygenase. The results of transcriptome showed that the expression level of the cytochrome P450 (*adhP*) gene encoding xenobiotic metabolism increased by 2.73-fold than that of the control group (Supplementary Table S3), which might be involved in the demethylation reaction of strain SL-6 to diuron. The gene expressions of N-formylglutamate amidohydrolase (I6I48_RS16595), amidohydrolase family (I6I48_RS20465), amidohydrolase family protein (I6I48_RS04995), and N-acetylmuramyl-L-alanine amidase (I6I48_RS09150) were significantly up-regulated (Supplementary Table S3), indicating that they could be involved in the hydrolysis of amide bonds and act as hydrolases to catalyze the cleavage of urea bridges in this process. The gene expression of 4-chlorobenzoyl-CoA dehalogenase (I6I48_RS07180) was up-regulated by 2.20-fold than that of the control group (Supplementary Table S3), which might be related to the reductive dechlorination reaction during the biodegradation of diuron. The gene expression of nitrate monooxygenase (I6I48_RS21595) was up-regulated by 2.35-fold than that of the control group (Supplementary Table S3), which could be relevant to the deamination oxidation reaction of aniline during the biodegradation of diuron. The expression of genes encoding multiple dioxygenases was significantly up-regulated, including quercetin 2,3-dioxygenase (I6I48_RS17155), aromatic ring hydroxylating dioxygenase (I6I48_RS26030), homogentisate 1,2-dioxygenase (*hmgA*) and phytanoyl-CoA dioxygenase family protein (I6I48_RS04300) (Supplementary Table S3), indicating that they might be responsible for ring-opening cleavage. Therefore, we speculate that there was a diuron degradation system composed of genes encoding multiple degradation enzymes in the strain SL-6 cells, which helped the strain SL-6 cells degrade and utilize diuron.

## 4 Discussion

The analysis of diuron degradation products provided information on the degradation pathway for the biodegradation mechanism of diuron by bacterial SL-6 cells. Previous studies indicated that microorganisms first generate DCPMU and DCPU by removing one or two methyl groups on diuron (Ellegaard-Jensen et al., 2013). This was followed by the hydrolysis of the amide bond, generating the metabolite 3,4-DCA, which was the common microbial diuron degradation product (Egea et al., 2017). According to previous reports, the biodegradation of 3,4-DCA mainly included two diverse reactions (dehalogenation and hydroxylation), followed by the formation of 4-chloroaniline (4-CA) and 1,2-dichlorobenzene (1,2-DCB) (Silambarasan et al., 2020). 4-CA was dechlorinated and hydroxylated to generate aniline and 4-chlorocatechol respectively, and then, aniline was deaminated to generate catechol (Mohlam et al., 2018; Li et al., 2022). Ultimately, ring-opening cleavage of catechol produced *cis, cis*-muconic acid (Shahryari et al., 2018; Yu et al., 2020). According to the seven degradation products identified by GC-MS, the degradation pathway of diuron in the cells of strain SL-6 was clarified. Mainly demethylation, urea bridge cleavage, dehalogenation, deamination and ring opening, which provided a basis for subsequent research on the biodegradation mechanism of diuron by strain SL-6.

Metabolomics was a science that reflects the physiological state of organisms by detecting changes in endogenous metabolites (Liang et al., 2021). Through metabolomic research, it was found that strain SL-6 requires the joint participation of the TCA cycle, glutathione metabolism, and urea cycle to adapt to and degrade diuron. As the center of metabolic pathways, the TCA cycle provided energy for other metabolic pathways in organisms (Liu et al., 2017; Ke et al., 2018). The degradation products of diuron could be utilized by SL-6 cells through the TCA cycle. Importantly, the TCA cycle could also produce organic acids to biosynthesize other amino acids (Arnold and Finley, 2023). These newly generated amino acids could participate in the regulation of energy metabolism, signaling pathways, and antioxidant defense responses in cells (Nesterov et al., 2020). The up-regulated arginine, glutamic acid, methionine, isoleucine, valine, proline, and threonine could directly or indirectly be involved in glycolysis, fat metabolism, pyruvate metabolism, and the TCA cycle, providing various nutrients for strain SL-6. Furthermore, Previous studies reported that glutathione was a common antioxidant in organisms and played an important role in maintaining cellular redox homeostasis, regulating cellular signals, and improving protein activity (Hasanuzzaman et al., 2017). Therefore, we speculated that the glutathione metabolic pathway could continuously provide antioxidants for SL-6 strain cells. The purpose was to remove the excessive ROS induced by diuron and maintain the redox homeostasis of SL-6 cells. Previous studies reported that arginine serves as an intermediate in the urea cycle, which could relieve ammonia poisoning and involved in the energy metabolism and antioxidant stress response of various microorganisms (Charlier and Bervoets, 2019; Hirose et al., 2021). Therefore, we speculate that the ammonia (NH_3_) removed during the degradation of diuron might eventually be excreted by the strain SL-6 cells via the urea cycle to achieve detoxification. Metabolomic analysis helped us to further expand our understanding of the mechanisms of degradation and adaptation of strain SL-6 to diuron.

Transcriptomics studies had shown that in order to fully degrade diuron, strain SL-6 cells might have cell transmembrane transport system, antioxidant system, and degradation system composed of a variety of different coding genes. Previous research had confirmed that most of the transmembrane transport of exogenous substances was mediated by the membrane transport system (Wu et al., 2023). Lipopolysaccharide, outer membrane protein, ABC, and MFS transporters were components of the cell transmembrane transport system. Studies had shown that lipopolysaccharide could adjust the hydrophobicity of bacterial cell surfaces and play an important role in the adsorption of organic contaminants (Okuda et al., 2016). Outer membrane proteins (OMPs) were structural proteins on the outer membrane of bacterial cells, which could participate in the passive and active transport of substances (Sun et al., 2022). It has been reported that ABC transporters and MFS transporters play important roles in cell transmembrane nutrient absorption (Mutanda et al., 2022). In addition, ABC and MFS transporters participated in the excretion of various toxic compounds within microbial cells and provided microorganisms with resistance to various compounds (Wilkens, 2015; Pasqua et al., 2021). We considered that diuron could enter the strain SL-6 cells through the cell transmembrane transport system to achieve multi-step degradation of diuron.

At present, research has shown that there was an endogenous antioxidant system in organisms, which could counteract free radicals and neutralize oxidants to maintain cellular redox homeostasis (He et al., 2017). The common antioxidants include catalase, peroxidase, glutathione metabolism, and redox homeostasis genes. It was reported that catalase and peroxidase in cells participate in the scavenging of ROS and reduce cytotoxicity (Hasanuzzaman et al., 2017). Glutathione S-transferase and glutaredoxin mediate the reduction of glutathione and were involved in the detoxification of xenobiotics and oxidative stress response (Dasari et al., 2018; Chai and Mieyal, 2023). Furthermore, genes related to cellular redox homeostasis play an important role in maintaining the normal physiological activities of cells and responding to oxidative stress (He et al., 2017). We considered that the genes encoding catalase, peroxidase, glutathione metabolism, and redox homeostasis together constitute the antioxidant system, which could help the strain SL-6 better adapt to the toxicity of diuron and provide a healthy and stable cell environment for the degradation of diuron in the cell.

In addition, the biodegradation of diuron mainly involved demethylase, amidase, amidohydrolase, dehalogenase, monooxygenase, and dioxygenase, which played different roles in the biodegradation of diuron. Demethylation reaction: the DCPMU and DCPU generated by diuron degradation indicate that the enzyme catalyzes the sequential demethylation of diuron (Silva Moretto et al., 2019). Cytochrome P450 was proved to participate in the demethylation of diuron by *Arthrobacter* N2 (Giacomazzi and Cochet, 2004). It was also involved in the demethylation reaction of diuron in humans and other mammals (Abass et al., 2007; Uno et al., 2011). Amide bond hydrolysis reaction: the most important enzymatic reaction of diuron biodegradation was the hydrolysis and cleavage of the amide bond to produce the metabolite 3,4-DCA (Hussain et al., 2015; Silambarasan et al., 2020). It was found that amidohydrolases and amidases played a role in the hydrolysis of amide bonds during the biodegradation of phenylurea herbicides. Seven species were reported, including PuhA, PuhB, LibA, HylA, Phh, Mhh, and LahB (Dejonghe et al., 2003; Khurana et al., 2009; Bers et al., 2012; Zhang et al., 2018, 2020).

Dechlorination reaction: the literature states that the next step after the biodegradation of 3,4-DCA was the sequential dechlorination reaction, which resulted in the production of aniline (Hongsawat and Vangnai, 2011). Dehalogenases could catalyze the removal of halogen groups and then were used by many bacteria to degrade halogenated aromatic hydrocarbons (Ang et al., 2018; Pimviriyakul et al., 2020). Therefore, the gene encoding dehalogenase might be involved in the reductive dechlorination reaction during the biodegradation of diuron. Oxidative deamination reaction: it has been reported that aniline was converted into catechol by deamination and hydroxylation (Arora, 2015). Monooxygenases could catalyze the deamination and oxidation of aromatic nitrogen oxides to generate hydroxylated products (Paul et al., 2021). The gene encoding monooxygenase might be involved in the biodegradation of diuron by strain SL-6 and play a role in the deamination of aniline to catechol. Ring-opening reaction: catechol could be further degraded into *cis, cis*-muconic acid (MA) through the ortho-cleavage pathway (Liu et al., 2023). According to various studies, dioxygenase had the ability to catalyze the ring-opening cleavage reaction of catechol (Zhang et al., 2021). The generated *cis, cis*-muconic acid was ultimately metabolized through the TCA cycle (He et al., 2023). We considered that there was a diuron degradation system composed of genes encoding demethylase, amidase, amidohydrolase, dehalogenase, monooxygenase, and dioxygenase in strain SL-6 cells, which helped the strain SL-6 cells to degrade and utilize diuron step by step, and finally realize the complete mineralization of diuron.

In summary, the biodegradation and adaption mechanisms of *Achromobacter xylosoxidans* SL-6 to diuron were further revealed through toxicological assessment, identification of intermediate metabolites, metabolome, and transcriptome analysis. Toxicological evaluation results showed that diuron could activate the antioxidant system (superoxide dismutase, catalase, and glutathione) in the cells of strain SL-6, and relieve the oxidative damage induced by a large number of reactive oxygen species. The analysis of degradation metabolites indicated that the diuron underwent a sequence of reactions in strain SL-6 cells, urea bridge cleavage, dehalogenation, deamination oxidation, and ring-opening. Metabolomic analysis revealed that the degradation of diuron by strain SL-6 mainly involved four metabolic pathways: diuron biodegradation, tricarboxylic acid cycle, glutathione metabolism, and the urea cycle. Furthermore, transcriptome data analysis identified differentially expressed genes that participated in substance transmembrane transport, antioxidant response, and diuron degradation in strain SL-6 cells. Overall, our research results offered a new perspective on the biodegradation and adaptation mechanism of strain SL-6 to diuron and promoted the environmental remediation strategy to a deeper level.

## Data availability statement

The original contributions presented in the study are publicly available. This data can be found here: NCBI Sequence Read Archive (SRA) database, accession number: PRJNA1118467.

## Author contributions

ZH: Data curation, Investigation, Software, Writing – original draft. CQ: Conceptualization, Writing – review & editing. HW: Conceptualization, Writing – review & editing. LS: Investigation, Writing – review & editing. CaW: Conceptualization, Funding acquisition, Methodology, Writing – review & editing. GZ: Resources, Supervision, Writing – review & editing. XH: Resources, Supervision, Writing – review & editing. ChW: Resources, Supervision, Writing – review & editing. TM: Resources, Supervision, Writing – review & editing. DY: Data curation, Project administration, Supervision, Writing – review & editing.
