# Supplementary material for: Comprehensive toxicological, metabolomic, and transcriptomic analysis of the biodegradation and adaptation mechanism by Achromobacter xylosoxidans SL-6 to diuron
Source: Front Microbiol. 2024 Jun 7;15:1403279. doi: 10.3389/fmicb.2024.1403279 (PMC11192067; doi:10.3389/fmicb.2024.1403279)
Supplement: Supplementary file 1 [file Data_Sheet_1.PDF]

Supplementary Material

1 Supplementary Data

Supplementary Section SI-1. Determination of the cytotoxicity of diuron to the strain SL-6

DCFH-DA (2 7-Dichlorofuorescin Diacetate) probe was by far the most commonly used and sensitive indicator for detecting intracellular reactive oxygen species (ROS). Malondialdehyde (MDA) usually reflected the degree of intracellular lipid peroxidation and was used to detect cell damage. Lactate dehydrogenase (LDH) activity could reflect cell membrane permeability and indicate the cytotoxicity of drugs. The above indicators were measured using kits from Nanjing Jiancheng Bioengineering Institute, and the measurement methods were carried out according to the manufacturer's instructions.

Supplementary Section SI-2. Analysis of diuron and its biodegradation products

Metabolites were analyzed using the MassHunter system and an Agilent gas chromatograph quadrupole mass spectrometer (Agilent 8890-5977B, Agilent, USA). An Agilent 19091S-433UI-INT column (30 m × 250 μm × 0.25 μm) was used, with helium as the carrier gas and a flow rate of 1 mL/min. The injection volume was 1 microlitre dichloromethane (100 %). The column temperature program starts at 40°C, held for 4 minutes, then heated the oven to 250 °C at 15 °C/min, held for 5 minutes, then heated to 300°C at 25°C/min, held for 10 minutes. Mass spectrometric analysis was performed in positive (ESI+) and negative (ESI−) modes using an electrospray ionization (ESI) source with a scan range from m/z 50–650. Data were collected and integrated using MassHunter 8.0 (Agilent, USA). The structures of compounds discovered during the degradation of diuron were drawn using KingDraw 5.0.

Supplementary Section SI-3. Metabolomic analysis

Metabolites from bacterial cells were extracted using methanol and acetonitrile. The sample was separated using an Agilent 1290 Infinity LC ultra-high performance liquid chromatography system (UHPLC), using a Waters ACQUITY UPLC BEH Amide column (2.1 mm × 100 mm column, 1.7 μm), with a column temperature of 25°C and a flow rate of 0.5 mL/min. The sample volume was 2 microlitre. Mobile phase composition A: water + 25 mM ammonium acetate + 25 mM ammonia water, B: acetonitrile. The gradient elution program was as followed: 0---0.5 min, 95% B; 0.5---7min, B changed linearly from 95% to 65%; 7---8 min, B changed linearly from 65% to 40%; 8---9 min, B was maintained at 40%; 9---9.1 min, B changed linearly from 40% to 95%; 9.1---12 min, B was maintained at 95%.

After the samples were separated using an Agilent 1290 Infinity LC ultra-high performance liquid chromatography system (UHPLC), mass spectrometry analysis was performed using a Triple TOF 6600 mass spectrometer (AB SCIEX), using electrospray ionization (ESI) positive ion and negative ion modes for detection. The ESI source setting parameters were as followed: atomization gas auxiliary heating gas 1 (Gas1): 60, auxiliary heating gas 2 (Gas2): 60, curtain gas (CUR): 30 psi, ion source temperature: 600°C, spray voltage (ISVF) ± 5500 V (positive and negative modes); first-level mass-to-charge ratio detection range: 60-1000 Da, second-level product ion mass-to-charge ratio detection

range: 25-1000 Da, first-level mass spectrometry scanning accumulation time: 0.20 s/spectra, The secondary mass spectrum scan accumulation time was 0.05 s/spectra; the secondary mass spectrum was obtained using data-dependent acquisition mode (IDA), and the peak intensity value screening mode was used. Declustering voltage (DP): ±60 V (positive and negative modes), Collision energy: 35±15 eV, IDA settings were as follows: Dynamic exclusion of isotope ions range: 4 Da, each scan collects 10 fragment spectra.

Supplementary Section SI-4. Transcriptome analysis

The strain was exposed to 200 mg/L diuron for 72 h, after which bacterial cells were collected by centrifugation (4 ℃) for RNA extraction. The total RNA was extracted and purified using the Total RNA Extraction Kit (Vazyme, Nanjing, China). The rRNA was removed using the ribo-Zero Magnetic Kit (Vazyme, Nanjing, China). The mRNA was cleaved into short fragments and then transcribed into cDNA to construct a cDNA library. The cDNA fragment library was sequenced by Illumina MiSeq, and the sequencing data were mapped to the reference sequence for gene expression analysis. The Fragments per Kilobase Million (FPKM) method was used to calculate gene expression levels. The DESeq software was used to identify differentially expressed genes (DEGs) according to the threshold of log2 Fold Change > 1 and p-value < 0.05. The DEGs were further mapped to the Kyoto Encyclopedia of Genes and Genomes (KEGG) and Gene Ontology (GO) database to determine their biological functions and metabolic pathways.

2 Supplementary Figures and Tables

2.1 Supplementary Figures

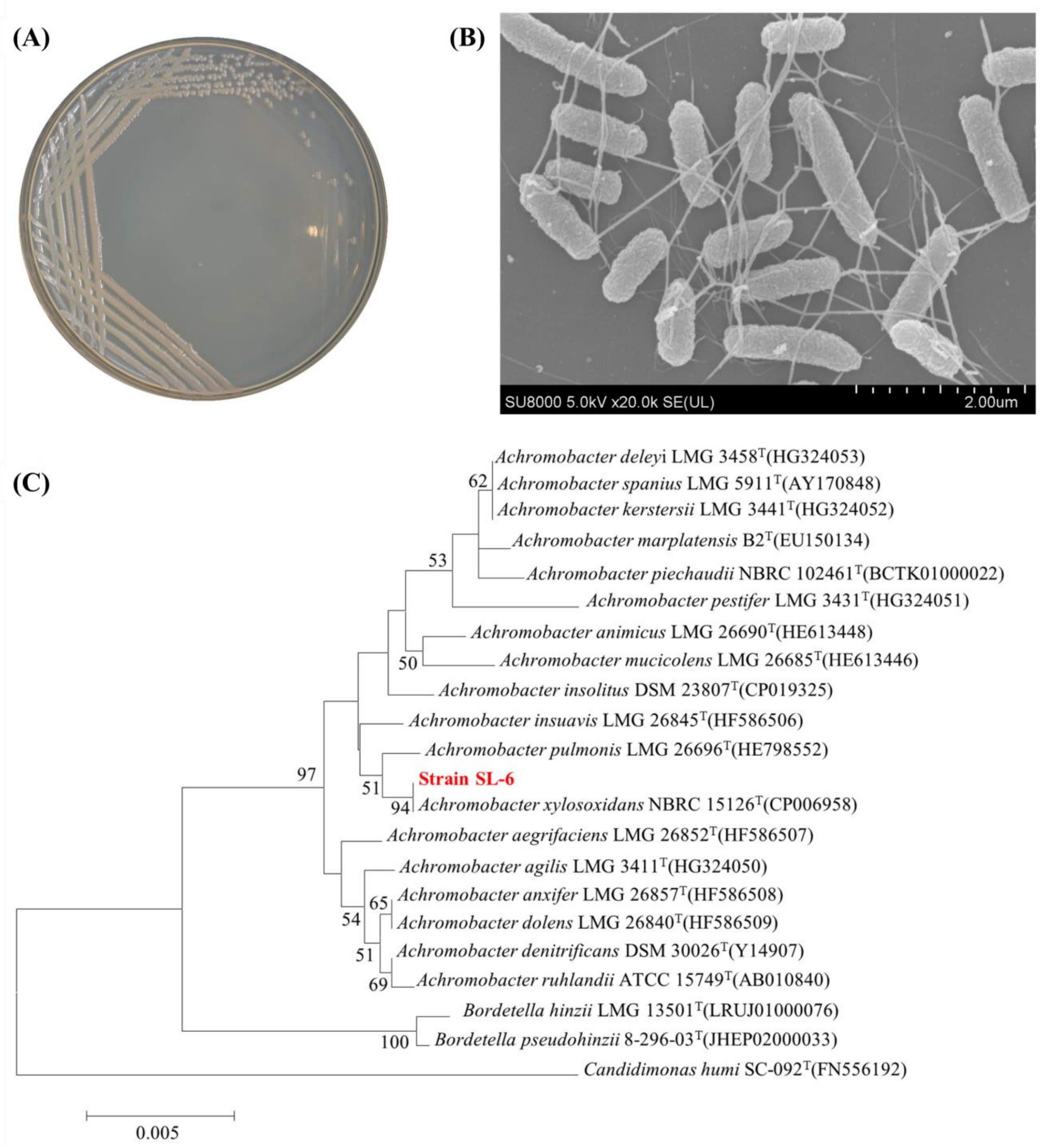

Supplementary Figure 1. (A) The colony morphology of *Achromobacter xylosoxidans* SL-6 on LB medium. (B) The SEM image of strain SL-6. (C) Phylogenetic tree constructed with 16S rRNA gene sequence using the nearest neighbor-joining method with 1000 bootstrap calculations, and *Achromobacter xylosoxidans* SL-6 in this study is underlined red.

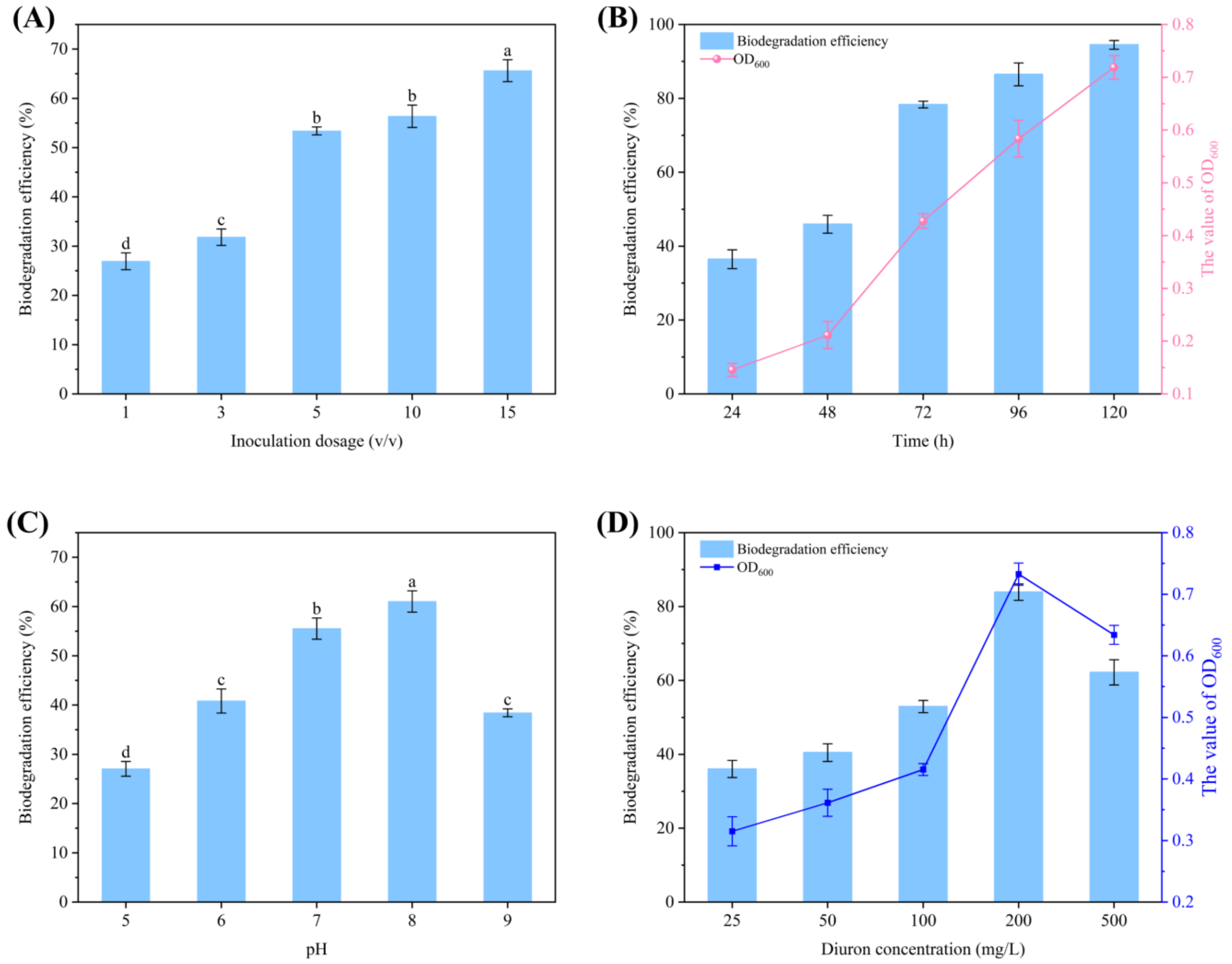

Supplementary Figure 2. (A) The effects of bacterial inoculum dosage, (B) biodegradation time, (C) pH, and (D) initial concentration of diuron on the efficiency of biodegradation of diuron by *Achromobacter xylosoxidan* SL-6. The right y-axis of b and d represented the growth of bacteria (OD_{600}).

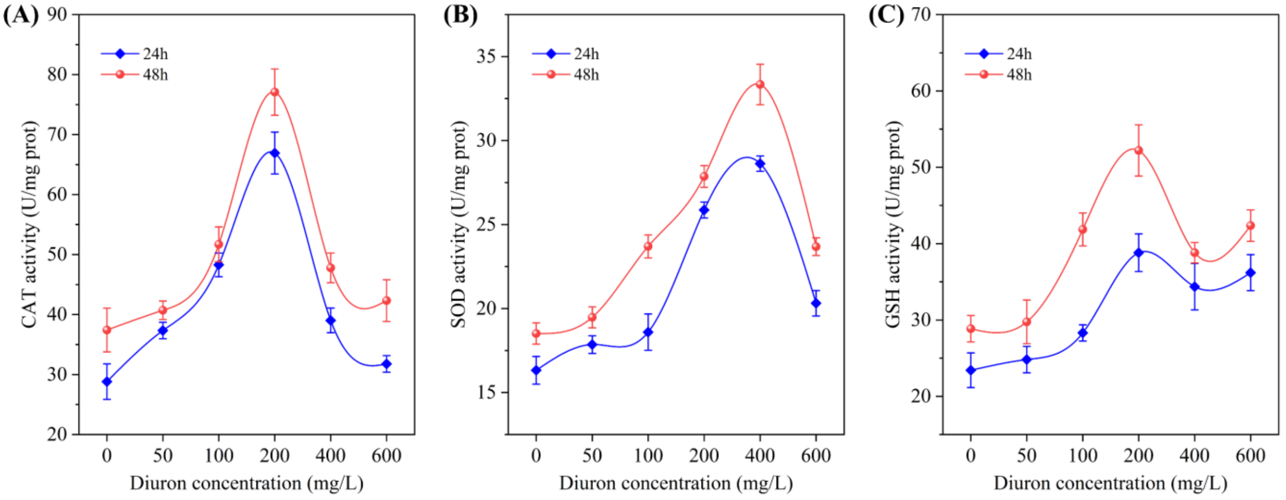

Supplementary Figure 3. Effects of different concentrations of diuron on (A) CAT activity, (B) SOD activity, and (C) GSH content of *Achromobacter xylosoxidans* SL-6 after 48 h treatment.

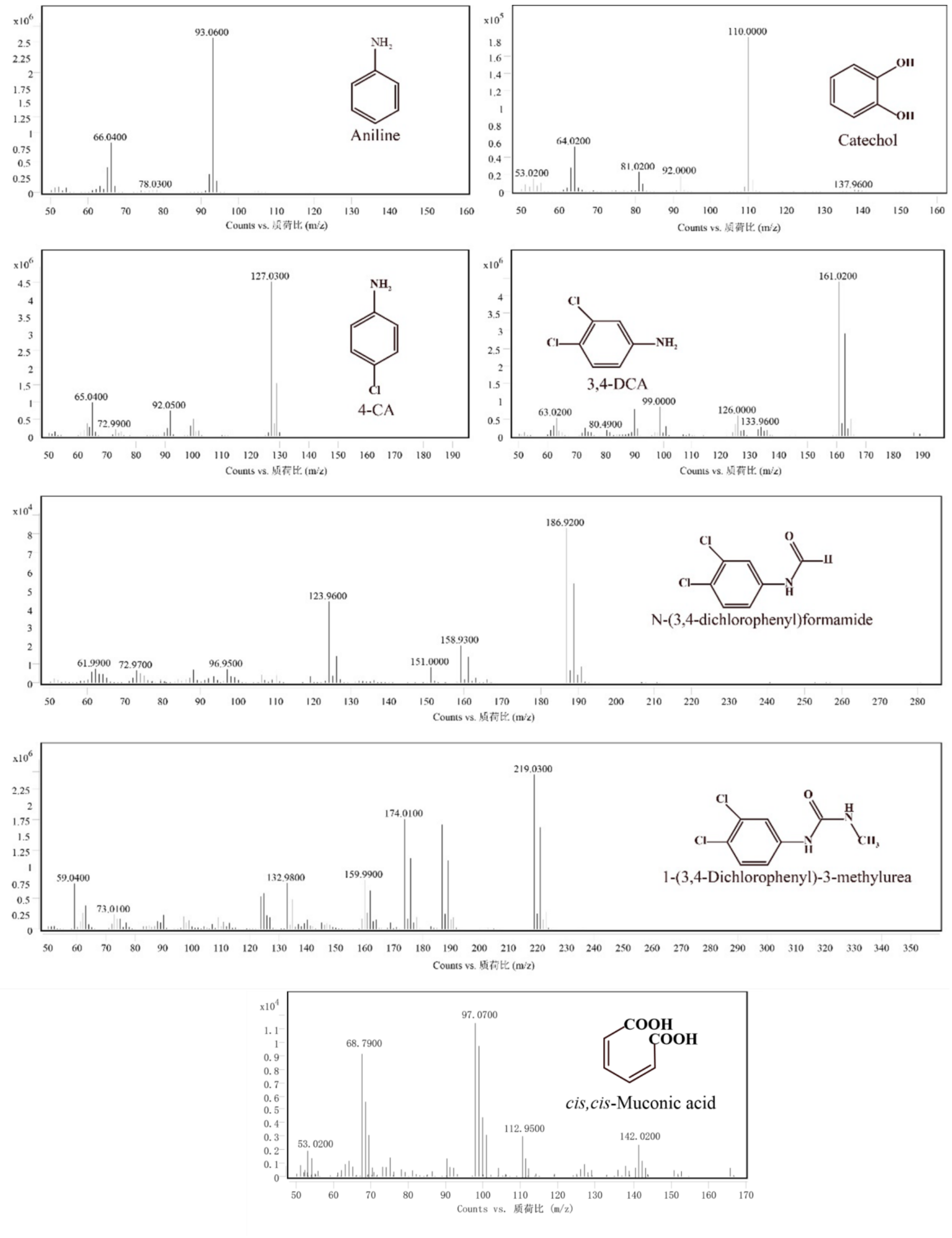

Supplementary Figure 4. GC-MS spectra and possible molecular structures of diuron biodegradation products identified by gas chromatography-mass spectrometry.

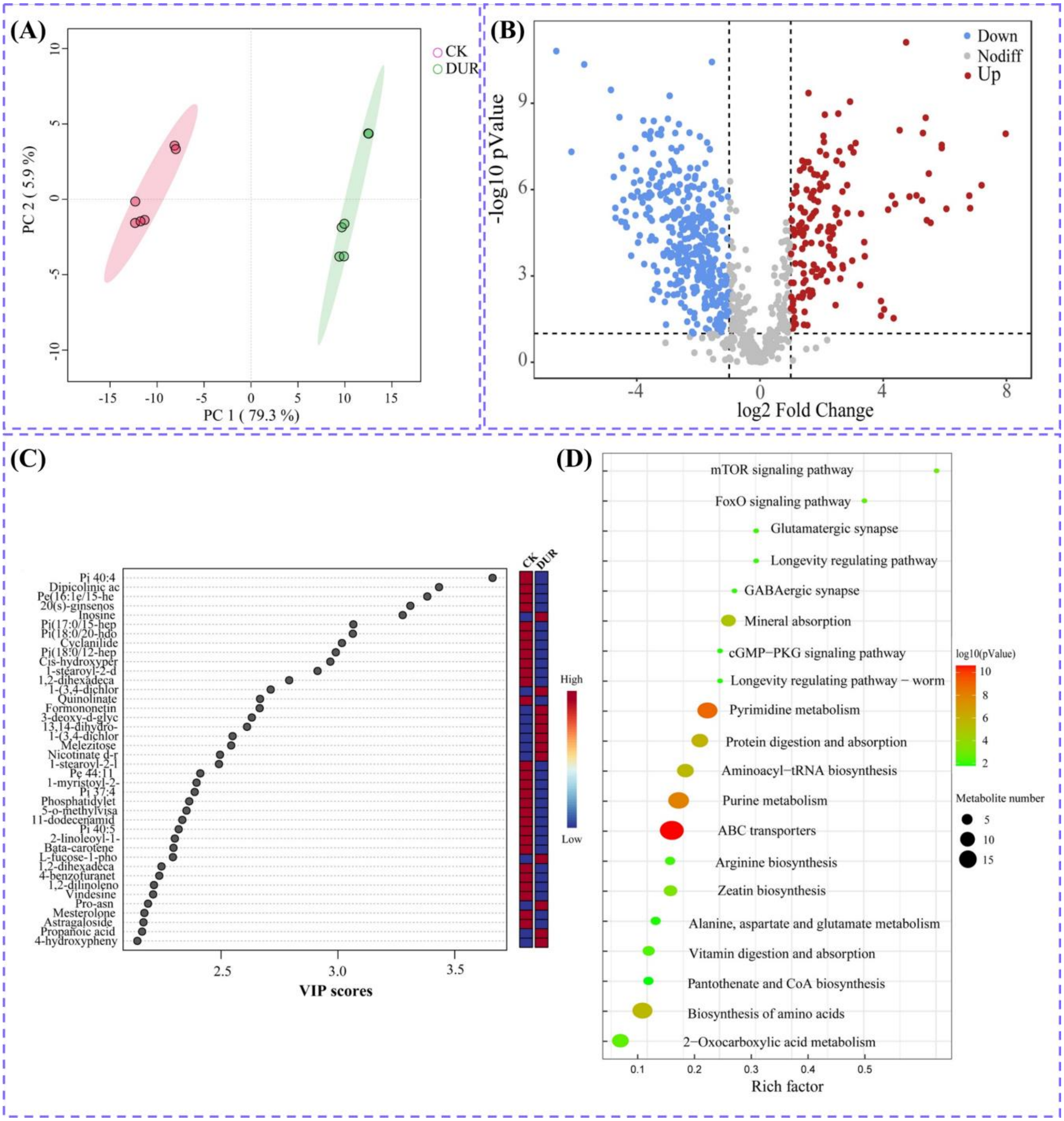

Supplementary Figure 5. Metabolomics analysis of strain SL-6 exposed to 200 mg/L diuron. (A) PCA plot. (B) Volcano plot of all identified metabolites. (C) VIP score plot. (D) KEGG pathway analysis of differential metabolites.

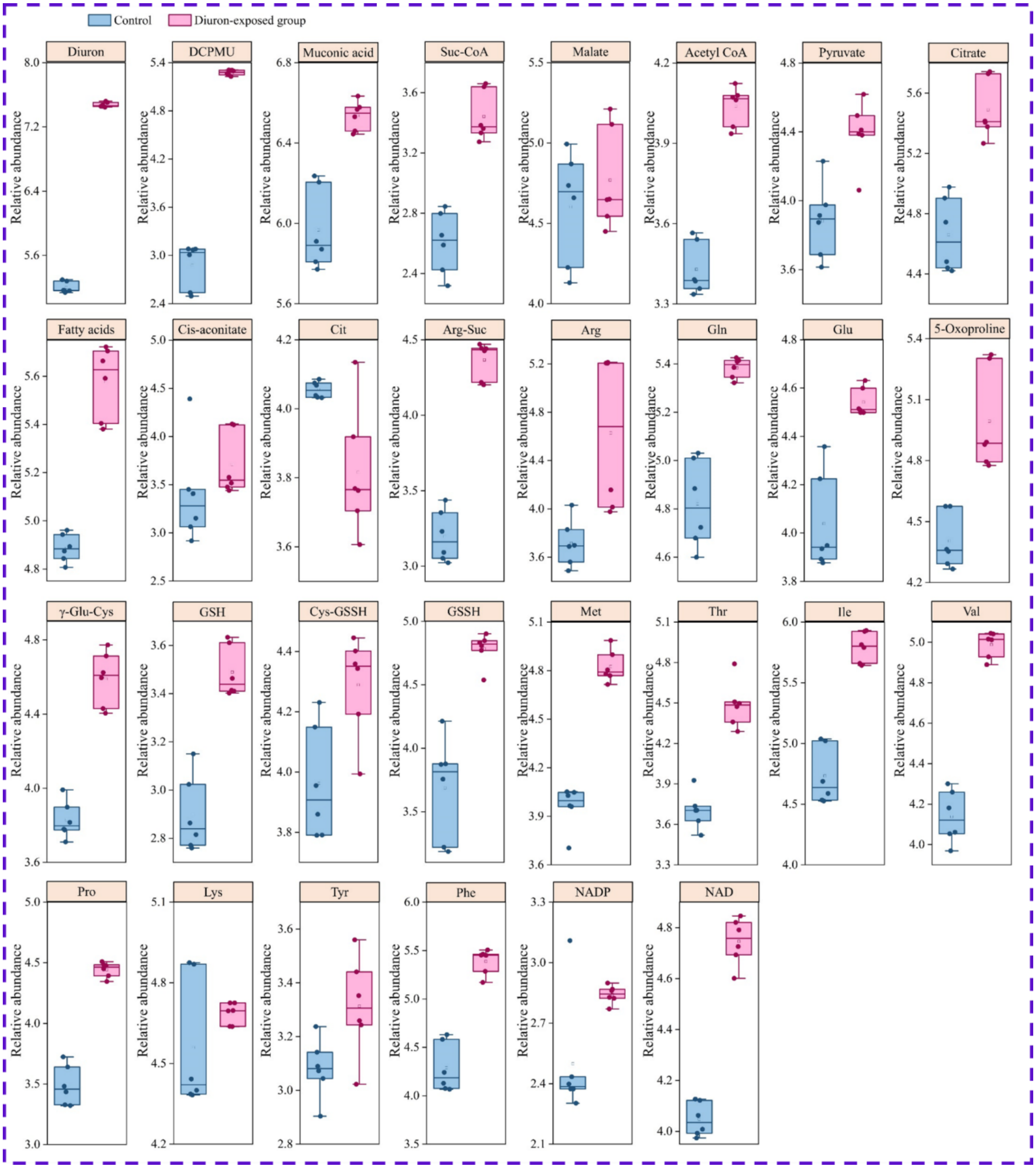

Supplementary Figure 6. Relative abundance of important metabolites related to the diuron biodegradation pathway, the TCA cycle, glutathione metabolism, and the urea cycle in strain SL-6.

2.2 Supplementary Tables

Supplementary Table 1. Differentially expressed genes related to transmembrane transport of substances in *Achromobacter xylosoxidans* SL-6 induced by diuron.

Gene ID	Up/down	log_2Fold Change	Fold Change	Description
I6I48_RS07135	Up	1.93	3.80	Lipopolysaccharide export system ATP-binding protein LptB
livG	Up	1.74	3.34	Lipopolysaccharide export system ATP-binding protein LptB
I6I48_RS11310	Up	1.59	3.01	Virulence-associated outer membrane protein Vir-90
adeC	Up	1.58	2.99	Outer membrane protein OprM
I6I48_RS00580	Up	1.68	3.20	Outer membrane protein OprM
I6I48_RS22785	Up	1.46	2.76	MFS transporter
I6I48_RS03180	Up	2.72	6.59	ABC transporter ATP-binding protein
I6I48_RS10650	Up	2.29	4.88	ABC transporter ATP-binding protein
dctP	Up	1.92	3.78	ABC transporter substrate-binding protein
I6I48_RS28500	Up	2.43	5.41	Amino acid ABC transporter substrate-binding protein
I6I48_RS29915	Up	1.73	3.31	Amino acid ABC transporter substrate-binding protein
I6I48_RS19805	Up	1.66	3.17	Branched-chain amino acid ABC transporter substrate-binding protein
I6I48_RS18970	Up	2.44	5.41	Carbohydrate ABC transporter substrate-binding protein
I6I48_RS23815	Up	1.50	2.83	C4-dicarboxylate ABC transporter
pstS	Up	1.72	3.28	Phosphate ABC transporter substrate-binding protein PstS

Supplementary Table 2. Differentially expressed genes related to antioxidant reactions and efflux pump in *Achromobacter xylosoxidans* SL-6 induced by diuron.

Gene ID	Up/down	Log_2Fold Change	Fold Change	Description
I6I48_RS30485	Up	1.31	2.48	Peroxidase activity
I6I48_RS29650	Up	1.33	2.51	Peroxidase activity
I6I48_RS11345	Up	1.25	2.39	Peroxidase activity
I6I48_RS02815	Up	1.48	2.80	Response to oxidative stress
I6I48_RS26100	Up	1.23	2.34	Glutathione S-transferase
I6I48_RS17070	Up	1.31	2.47	Glutaredoxin
I6I48_RS04185	Up	1.60	3.04	Catalase
I6I48_RS18035	Up	1.04	2.05	Cell redox homeostasis
I6I48_RS30485	Up	1.31	2.48	Cell redox homeostasis
I6I48_RS05390	Up	1.36	2.57	Cell redox homeostasis
I6I48_RS18020	Up	2.65	6.26	Efflux pump membrane transporter
I6I48_RS09355	Up	1.83	3.57	Efflux pump membrane transporter

Supplementary Table 3. Differential expression of degradation enzyme-related genes in *Achromobacter xylosoxidans* SL-6 induced by diuron

Gene ID	Up/down	log2Fold Change	Fold Change	Description
I6I48_RS05445	Up	2.76	6.77	Class I SAM-dependent methyltransferase
ubiE	Up	1.03	2.04	Methyltransferase
I6I48_RS16595	Up	1.29	2.45	N-formylglutamate amidohydrolase
I6I48_RS20465	Up	1.08	2.11	Amidohydrolase family
I6I48_RS04995	Up	1.08	2.11	Amidohydrolase family protein
I6I48_RS09150	Up	1.36	2.57	N-acetylmuramoyl-L-alanine amidase
I6I48_RS17155	Up	2.09	4.26	Quercetin 2,3-dioxygenase
I6I48_RS26030	Up	2.04	4.11	Aromatic ring hydroxylating dioxygenase
hmgA	Up	1.75	3.36	Homogentisate 1,2-dioxygenase
I6I48_RS04300	Up	1.17	2.25	Phytanoyl-CoA dioxygenase family protein
I6I48_RS21595	Up	1.23	2.35	Nitronate monooxygenase
adhP	Up	1.45	2.73	Metabolism of xenobiotics by cytochrome P_{450}
I6I48_RS17065	Up	2.05	4.14	Phenol degradation protein
I6I48_RS07180	Up	1.14	2.20	4-chlorobenzoyl coenzyme A dehalogenase
